# Editorial: Epigenetics in Mammalian Tissues

**DOI:** 10.3389/fgene.2019.00635

**Published:** 2019-07-05

**Authors:** Rafael Franco, Michael E. Symonds

**Affiliations:** ^1^Department Bioquimica i Biomedicina Molecular, Universitat de Barcelona, Barcelona, Spain; ^2^CIBERNED, Centro de Investigación en Red, Enfermedades Neurodegenerativas, Instituto de Salud Carlos III, Madrid, Spain; ^3^Early Life Research Unit, Division of Child Health, Obstetrics and Gynaecology, and Nottingham Digestive Disease Centre and Biomedical Research Centre, School of Medicine, University of Nottingham, Nottingham, United Kingdom

**Keywords:** cytocrin, Turner syndrome (TS), X chromosome, epigenetic abnormalities, DNA methylation

The challenge of understanding epigenetics and reaching a consensus for its role(s) in development is substantial. This is illustrated in the wide range of topics covered by the research topic “Epigenetics in Mammalian Tissues.” The discovery of new concepts in epigenetics during pregnancy and early development is on-going, together with their potential impact on susceptibility to later disease. A diverse range of examples are included within this issue that cover methodological approaches together with responses of individual tissues and organs ranging from the placenta to the eye.

The paper focused on the eye shows evidence of epigenetic factors in diseases affecting organs involved in sense perception. This could be important because in contrast to genetic inheritance of disease, epigenetic factors may be acquired by a variety of circumstances. In the case of the eye, adverse exposure to sunlight can trigger a range of responses that can contribute to the onset of cataracts to glaucoma (Alkozi et al.).

It is fascinating that cells with the same DNA content may give rise to such diverse tissues, and epigenetic factors are important in this regard. Undoubtedly, development and function of the central nervous system (CNS) is under epigenetic modification, as it is composed of a myriad of phenotypically different cells. For example, whereas the liver is primarily composed of hepatocytes and fibroblasts, the CNS contains neurons and glia with hundreds of diverse neurons. One paper in the special issue focuses on the epigenetic processes taking place in the CNS and how this impacts on the nuclear architecture during development, function, and disease onset (Ito and Takizawa).

Another paper describes a study (He et al.) using a transgenic mouse to model Turner disease, which is a chromosomal disease characterized “by complete or partial X monosomy in some or all cells” (Saenger et al., 2001). It compares mice with one single paternally or maternally inherited X chromosome, of which embryos with paternal inheritance have greater risk of miscarriage (Hall and Gilchrist, 1990; Robinson, 1990). This is probably due to epigenetic factors such as paternally imprinted genes in the placenta or genome-wide differences in chromatin condensation. Differences in the outer zone of the placenta occupied by glycogen cells together with a substantial adaptation of glycogen/glucose storage and metabolism are described. They conclude that changes in glucose metabolism are not directly caused by changes in X-linked genes but are more likely to be a consequence of an altered cellular composition mediated by placental adaptation in early development.

The study of the impact of the X chromosome on health and disease is fascinating. One aspect relates to *in vitro* fertilization, and (Min et al.) suggest that epigenetic factors could be involved in the differential expression of X-linked genes in male compared with female *in vitro* fertilization-derived blastocysts. They also demonstrate epigenetic regulation of X chromosome reactivation with somatic-cell nuclear transfer. Under these conditions, clonal reprogramming of X-chromosome appears incomplete and may differently occur in cells within a single blastocyst.

There are a wide range of methods available for examining epigenetics including the assessment of patterns of DNS methylation, histone acetylation, etc. The paper by Jeon et al. is technical in nature and describes how it is possible to run parallel assessments of methylation status in hundreds of CpG sites. Their approach is based on bisulfite polymerase chain reaction sequencing (TBPseq). The field has also advanced by exploring how to enhance or depress methylation/acetylation as a therapeutic approach. For example, inhibition of histone acetylation leads to an increase in the expression of genes that enhance cognition and could have potential in treating Alzheimer’s disease (Cuadrado-Tejedor et al., 2013; Cuadrado-Tejedor et al., 2015).

It should also be noted the term epigenetic is not used by all scientists in the same way. One article revisits the concept that epigenetics defines everything that is not genetic, i.e., existing in the DNA sequence, that affects phenotype. On doing so, hormonal regulation, that ultimately impacts on gene expression, could be considered epigenetic. This reasoning makes it necessary to choose a word to define events occurring inside the cell, i.e., those participating after a hormone interacts with a cell surface receptor until a transcription factor is activated or inactivated and gene expression is enhanced or repressed. The word used by Navarro et al. has the prefix “cyto” and the suffix “crin,” as there are interacting intracellular cascades with actions at the nuclear level. Specific cytocrin pathways may explain the differential effect of any hormone on mammalian tissues or cells thereby linking epigenetic factors and epigenetic mediators to epigenetic traits. This type of concept could explain the impact of modulating maternal nutrition for which supplementation of the maternal diet with fructose, in rats, modulates the maternal microbiome (Astbury et al.) and it was accompanied with reduced gene expression of markers of gut barrier function that could adversely affect its function in later life.

In conclusion, our understanding of epigenetic regulation of mammalian tissues is only beginning to become established. Furthermore, as illustrated by the diverse nature of papers included in this edition, the topic has substantial scope for transforming our understanding of development and disease onset.

## Conflict of Interest Statement

The authors declare that the research was conducted in the absence of any commercial or financial relationships that could be construed as a potential conflict of interest.
